# Biomechanical comparison of pedicle screw fixation strength among three different screw trajectories using single vertebrae and one-level functional spinal unit

**DOI:** 10.3389/fbioe.2022.1054738

**Published:** 2022-12-09

**Authors:** Ching-Lung Tai, Weng-Pin Chen, Mu-Yi Liu, Yun-Da Li, Tsung-Ting Tsai, Po-Liang Lai, Ming-Kai Hsieh

**Affiliations:** ^1^ Department of Biomedical Engineering, Chang Gung University, Taoyuan, Taiwan; ^2^ Department of Orthopaedic Surgery, Spine Section, Bone and Joint Research Center, Chang Gung Memorial Hospital and Chang Gung University College of Medicine, Taoyuan, Taiwan; ^3^ Department of Mechanical Engineering, National Taipei University of Technology, Taipei, Taiwan

**Keywords:** pedicle screws, cortical bone trajectory, functional spinal unit, porcine model, screw pullout test

## Abstract

Three key factors are responsible for the biomechanical performance of pedicle screw fixation: screw mechanical characteristics, bone quality and insertion techniques. To the best of the authors’ knowledge, no study has directly compared the biomechanical performance among three trajectories, i.e., the traditional trajectory (TT), modified trajectory (MT) and cortical bone trajectory (CBT), in a porcine model. This study compared the pullout strength and insertion torque of three trajectory methods in single vertebrae, the pullout strength and fixation stiffness including flexion, extension, and lateral bending in a one-level instrumented functional spinal unit (FSU) that mimics the *in vivo* configuration were clarified. A total of 18 single vertebrae and 18 FSUs were randomly assigned into three screw insertion methods (*n* = 6 in each trajectory group). In the TT group, the screw converged from its entry point, passed completely inside the pedicle, was parallel to the superior endplate, was located in the superior third of the vertebral body and reached to at least the anterior third of the vertebral body. In the MT group, the convergent angle was similar to that of the TT method but directed caudally to the anterior inferior margin of the vertebral body. The results of insertion torque and pullout strength in single vertebrae were analyzed; in addition, the stiffness and pullout strength in the one-level FSU were also investigated. This study demonstrated that, in single vertebrae, the insertion torque was significantly higher in CBT groups than in TT and MT groups (*p* < 0.05). The maximal pullout strength was significantly higher in MT groups than in TT and CBT groups (*p* < 0.05). There was no significant difference in stiffness in the three motions among all groups. The maximal pullout strength in FSUs of MT and CBT groups were significantly higher than the TT groups (*p* < 0.05). We concluded that either MT or CBT provides better biomechanical performance than TT in single vertebrae or FSUs. The lack of significance of stiffness in FSUs among three methods suggested that MT or CBT could be a reasonable alternative to TT if the traditional trajectory was not feasible.

## 1 Introduction

Pedicle screw fixation for lumbar spinal segments has been described for a variety of surgical indications including scoliosis, deformity, fracture, infection or tumors (Wang et al., 2014; Tschugg et al., 2017; Fan et al., 2018; Chan et al., 2020; Perna et al., 2022). Immediate stability of pedicle screw-rod instrumentation provides the benefit of a quicker and reliable fusion mass and finally reaches permanent stability (Wang et al., 2019; Zhao et al., 2019; He et al., 2020). Several factors affect the fixation stability of a pedicle screw, including the screw shape, diameter, length, thread type/shape, pitch width, outer/inner core difference and bone mineral density (Liu et al., 2020; Hsieh et al., 2021a). In addition to the morphometric characteristics of the pedicle screw, the trajectory of the screw also has a strong influence on fixation strength (Lehman et al., 2003; Phan et al., 2015; Delgado-Fernandez et al., 2017; Jarvers et al., 2021). Currently, three methods for the insertion of pedicle screws are widely used in the lumbar spine. First, in the traditional trajectory (TT), the screw converges from its entry point, passes completely inside the pedicle, and reaches at least the anterior third of the vertebral body (Youssef et al., 1999; Lehman et al., 2003; Phan et al., 2015; Wadhwa et al., 2015; Delgado-Fernandez et al., 2017; Jarvers et al., 2021). In the sagittal plane, traditional screws should be located in the superior third of the vertebral body and parallel to the superior endplate (Lehman and Kuklo, 2003; Suk et al., 2005). Second, the modified trajectory (MT) has a convergent angle similar to that of the TT but directed caudally to the anterior inferior margin of the vertebral body without penetrating the inferior endplate (Lehman et al., 2003; Lehman and Kuklo, 2003; Wadhwa et al., 2015). Anatomically, longer-sized screws could be used in MT, which is supposed to purchase more bone than the traditional method (Hsieh et al., 2019; Hsieh et al., 2020). Third, cortical bone trajectory screws (CBT), starting medially from the pars inter-articularis and following a cranio-lateral direction through the pedicle with the objective of maximizing thread contact with cortical bone, have been proven to have similar clinical outcomes and better operative parameters, such as shorter incision length, quicker operative time, and less blood loss, than TTs in posterior lumbar interbody fusion surgery (Perez-Orribo et al., 2013; Lee et al., 2015; Oshino et al., 2015). However, inconsistent biomechanical results among different trajectories come from using different bone densities, different species of cadaveric specimens (Perez-Orribo et al., 2013; Lee et al., 2015; Oshino et al., 2015; Delgado-Fernandez et al., 2017), various finite element models (Matsukawa et al., 2017; Molinari et al., 2021), uncontrolled cephalad angles of CBT screws (Perez-Orribo et al., 2013; Oshino et al., 2015), and undefined MT axes (Lehman et al., 2003; Lehman and Kuklo, 2003). Studies to date have been predominantly based on single screw fixation stability, which may not be the actual clinical representation of implant failure (Lehman et al., 2003; Lehman and Kuklo, 2003; Delgado-Fernandez et al., 2017; Jarvers et al., 2021). To the best of our knowledge, no study has directly compared the biomechanical performance among these three trajectories in single vertebrae and one-level functional spinal units (FSUs) in a porcine model.

The purpose of the present study was to compare the pullout strength and insertion torque of three trajectory methods in single vertebrae; moreover, the pullout strength and fixation stiffness including flexion, extension, and lateral bending in one-level instrumented FSUs that mimicked the *in vivo* configuration were clarified to recommend further clinical use.

## 2 Materials and methods

This study was approved by the committee of Ministry of Science and Technology, Taiwan (MOST 109-2221-E-182-006-MY2). All specimens were purchased from commercial meat market (Yahsen Frozen Foods Co., Taiwan) and were exempted from filing an Institutional Animal Care and Use Committees (IACUCs) protocol for the use of dead animal-derived bone.

### 2.1 Specimen preparation and implantation

**FIGURE 1:**
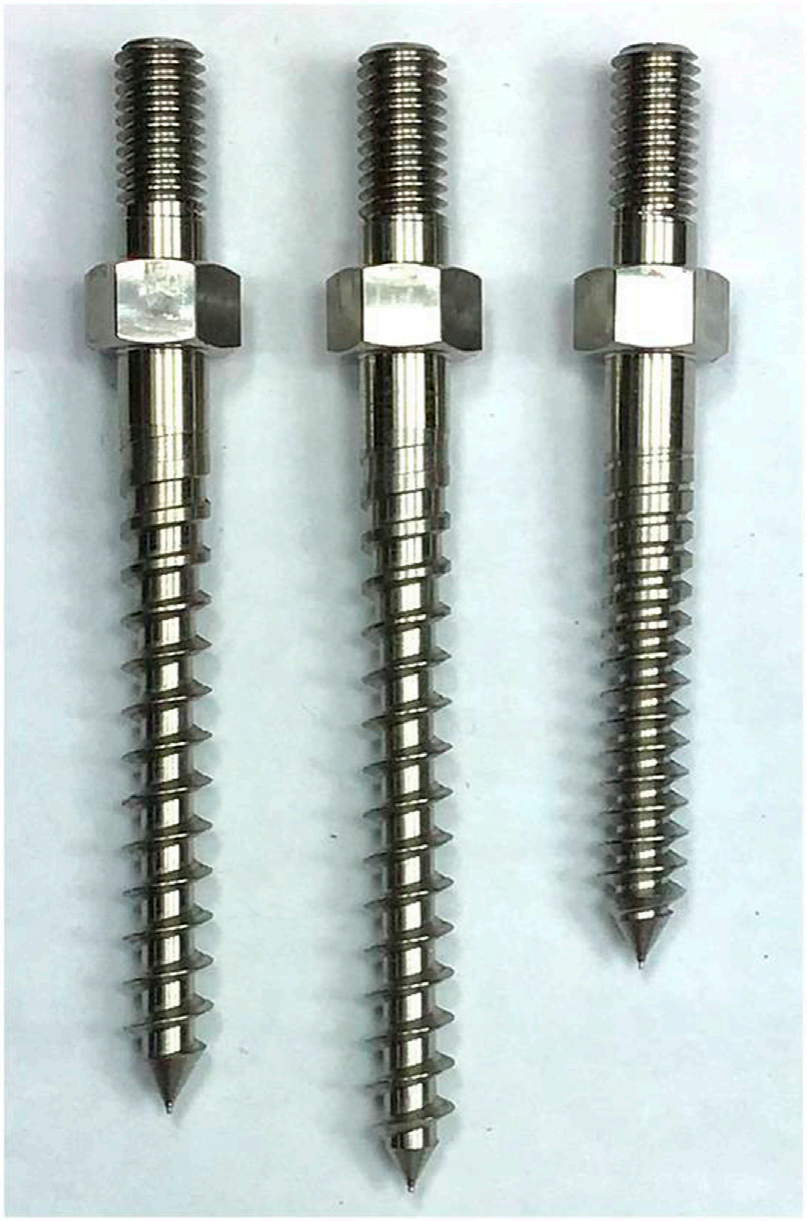
Photograph showing three types of pedicle screws used in TT, MT, and CBT trajectories (from left to right). The dimensions (diameter × length) of the TT, MT and CBT screws were 6.0 mm × 45 mm, 6.0 mm × 50 mm and 6.0 mm × 35 mm, respectively. The screw pitches of the TT, MT and CBT screws were 3.0 mm, 3.0 mm and 1.5 mm, respectively. All screws had identical thread depths of 1.2 mm.

**FIGURE 2:**
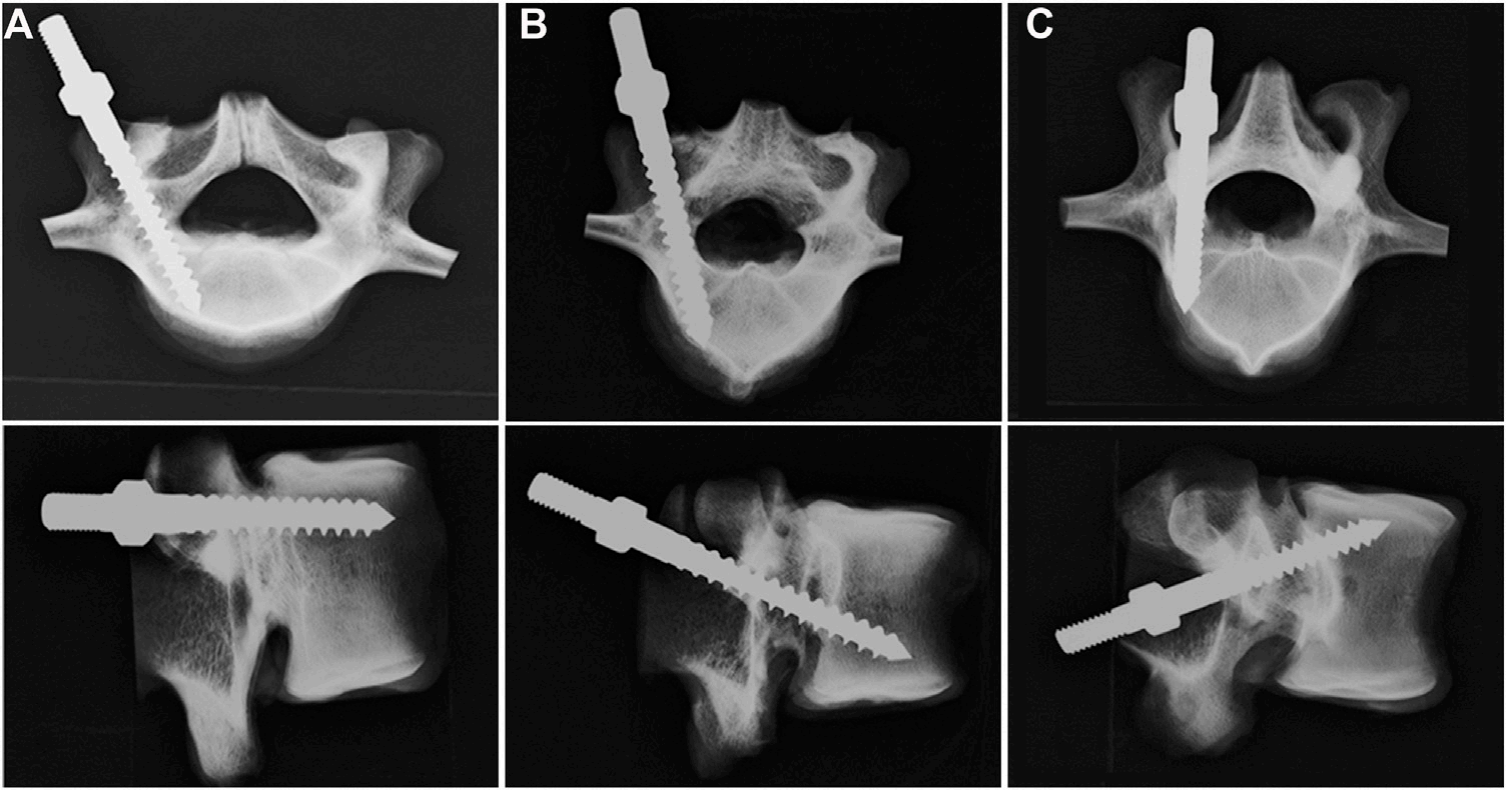
Axial (upper) and sagittal (lower) X-ray images of the vertebrae after the insertion of screws with **(A)** TT, **(B)** MT, and **(C)** CBT trajectories. In the axial view, the pedicle screws in the TT and MT groups were convergently inserted into the vertebral body, whereas in the CBT group, the screw was inserted divergently toward the lateral cortex. In the sagittal view, the screw in the TT group was parallel to the superior endplate; in the MT group, the screw was inserted toward the anterior inferior edge of the vertebral body with proper depth. In the CBT group, the screw was placed 25° caudally toward the superior endplate. No fractures or lateral wall breach were detected in either view.

**FIGURE 3:**
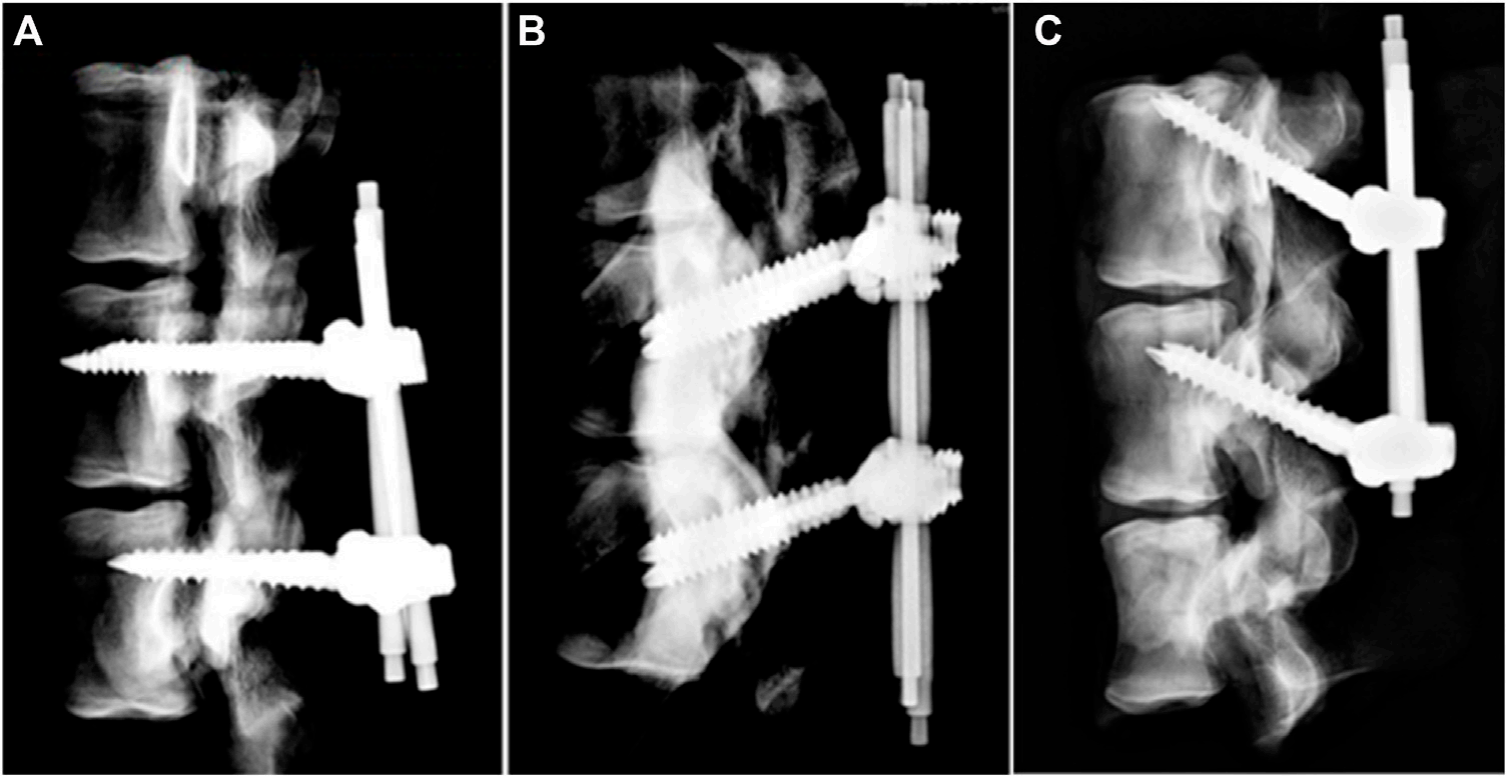
X-ray images of the FSU constructs after assembly of the screw and rod. **(A)** TT, **(B)** MT, and **(C)** CBT. Pedicle screws in each trajectory group of FSUs were implanted according to methods in single vertebra with paired segmental pedicle screws (diameter × length of 6.0 mm × 45 mm in the TT group, 6.0 mm × 50 mm in the MT group and 6.0 mm × 35 mm in the CBT group) and 5.5 mm diameter titanium rods were used to connect the pedicle screws in all three groups.

A total of 18 single vertebrae and 18 FSUs were used in the study, with L1-6 fresh-frozen lumbar vertebrae harvested from mature pigs (weight 100–110 kg). All animals were healthy before harvesting and never exposed to any drugs or treatments that could affect the bone mineral density. All the specimens were separated into individual vertebrae or FSUs after being stripped of the surrounding musculature, ligaments, and periosteum. All specimens were stored at -20°C until the day of testing and thawed for 24 h before implantation. A pilot hole was drilled using a 2.5 mm “twist” metric drill bit attached to a Dremel 4000 rotary tool that was mounted on a Dremel WorkStation Model 220–01. The pilot hole of the TT group was at the junction of the transverse process and facet joint. Drilling was determined using lateral fluoroscopy, and the direction was parallel to the superior endplate and medial to produce a converging appearance. The pilot track was followed with a 2.5 mm standard straight pedicle drill to a depth of 40 mm. Cylindrical coarse-thread screws with a size of 6.0 mm × 45 mm were inserted until the final thread was engaged in the lateral facet cortex. In the MT group, the pilot hole was at the superior edge of the superior articular process, and the pilot track was followed with a 2.5 mm standard straight pedicle drill to a depth of 45 mm toward the anterior inferior edge of the vertebral body under lateral fluoroscopy. Cylindrical coarse-thread screws with a size of 6.0 mm × 50 mm were inserted in this group. In the CBT group, the pilot hole in the pars inter-articularis was created using a 2.5 mm drill bit and followed a 25° caudal and 25° lateral trajectory to a pilot tract of 30 mm (Matsukawa et al., 2013). Cylindrical fine-thread screws with a size of 6.0 mm × 35 mm (Figure 1) were inserted in this group. In the 18 FSUs, polyaxial screws (Baui Biotech, Co., Taipei, Taiwan) were chosen and randomly implanted into each pedicle of the vertebrae by an experienced surgeon. Six FSUs in each trajectory group were implanted according to methods in single vertebra with paired segmental pedicle screws (diameter × length of 6.0 mm × 45 mm in the TT group, 6.0 mm × 50 mm in the MT group and 6.0 mm × 35 mm in the CBT group), and 5.5 mm diameter titanium rods were used to connect the pedicle screws in all three groups. Axial and sagittal views were examined *via* X-ray imaging for all specimens prior to the biomechanical test to confirm an appropriate screw trajectory and insertion depth in single vertebrae (Figure 2) and FSUs (Figure 3). The specimens were also examined thoroughly to rule out any fractures or defects caused by screw insertion.

### 2.2 Biomechanical testing

**FIGURE 4:**
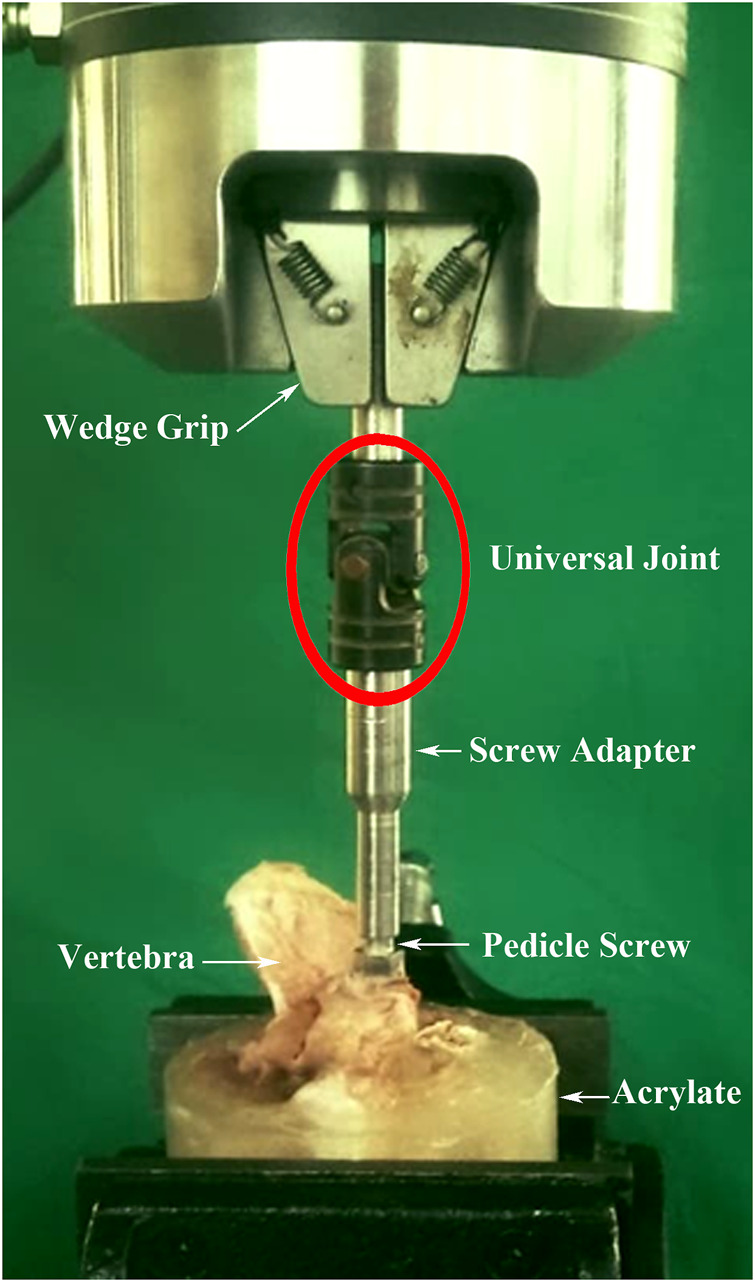
Experimental setup of the screw pullout test of the single vertebrae. The screw head was fixed to one end of an adapter having an inner thread that matched the outer thread of the screw head. The other end of the adapter was then clamped to the upper wedge grip of the MTS testing machine. The adapter equipped with a universal joint ensuring vertical pullout alignment during the pullout test.

In single vertebrae, the maximal insertion torque was measured with an electronic torque wrench (OLY 921/6NB, New Taipei City, Taiwan) in the last thread of screw insertion. Each of the 18 single instrumented vertebrae was embedded in acrylate resin (#20–3568; Buehler, Lake Bluff, IL, United States) to allow clamping during the screw pullout test. The method for the screw pullout test was identical to that used in our previous study (Hsieh et al., 2019; Hsieh et al., 2020). Each prepared specimen was secured to a custom-made grip mounted on the platform of the testing machine (Bionix 810; MTS Systems Corp., MN, United States) to conduct axial pullout tests of the screws (Figure 4). The screw head was fixed to one end of an adapter having an inner thread that matched the outer thread of the screw head. The other end of the adapter was then clamped to the upper wedge grip of the MTS testing machine. The adapter equipped with a universal joint ensuring vertical pullout alignment during pullout test. The potted specimen was secured on a lower custom-made grip capable of x-y plane translation and rotation to achieve the coaxial alignment of the pedicle screw with the pullout arm. After the specimens were mounted, pullout force was applied at a constant crosshead rate of 5 mm/min. During the pullout test, the relation between the applied force and displacement was simultaneously recorded in 0.1 mm increments until failure. The peak force recorded was defined as the ultimate pullout strength for comparison.

**FIGURE 5:**
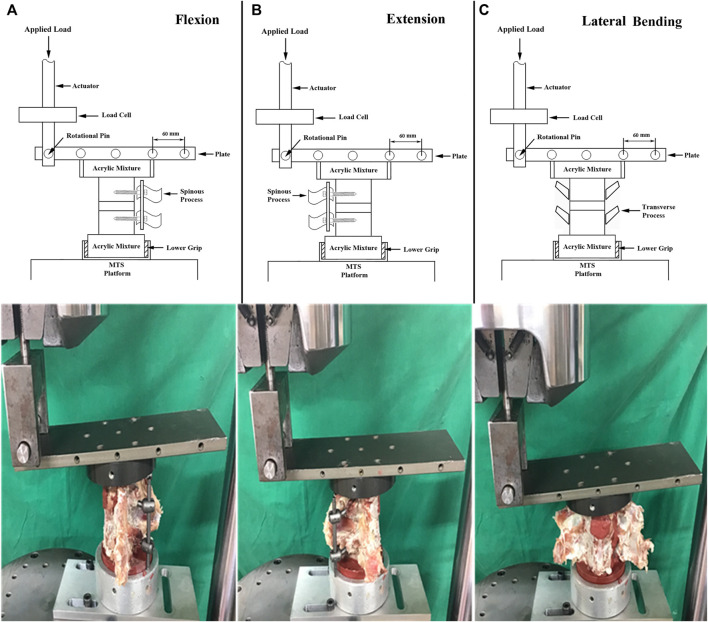
Schematic drawings (upper) and photographs showing the experimental setup for **(A)** flexion, **(B)** extension, and **(C)** lateral bending of the FSU constructs. The tests were performed on a biaxial (axial and rotation) MTS machine. A maximal 8,400 N-mm moment generated through the axial movement of the MTS actuator was applied to the spine specimen to achieve flexion, extension and lateral bending motions.

In the FSUs, the specimens were mounted for flexion, extension, and lateral bending using an axial MTS testing machine (Bionix 858, MTS Corp., MN, United States) (Tai et al., 2008; Hsieh et al., 2021b). The superior segment was embedded in acrylic resin (#20–3568; Buehler, Lake Bluff, IL, United States) and constrained by the upper clamp with an adjustable moment arm, whereas the inferior segment, embedded in acrylic resin, was constrained by the lower clamp (Figure 5). This experimental setup resulted in a compressive preload of 20 N due to the weight of the upper fixation acrylic resin. Each FSUs was non-destructively tested in three sequential modes: flexion, extension, and lateral bending. The clamp was designed with a pin that rotated horizontally across the upper plate, and the pin was perpendicular to the motion plane of the specimen. The horizontal pin and vertical motion path of the specimen resulted in a *3-D* configuration that ensured that the specimen moved vertically as the spinal construct was flexed, extended or laterally bent. The position of the horizontal pin was adjusted to set the moment arm to 120 mm, and an increasing compressive force up to 70 N was applied to the horizontal pin across the upper plate. Therefore, the resultant applied moment was 8,400 N-mm, which remains within the viscoelastic range (Karakaşli et al., 2013; Karakaşli et al., 2014). During testing, the displacement data associated with the applied moment were recorded simultaneously. The FSU stiffnesses in flexion, extension, and lateral bending were defined as the applied moment divided by the value of displacement at the latest stage for all three loading modes.

**FIGURE 6:**
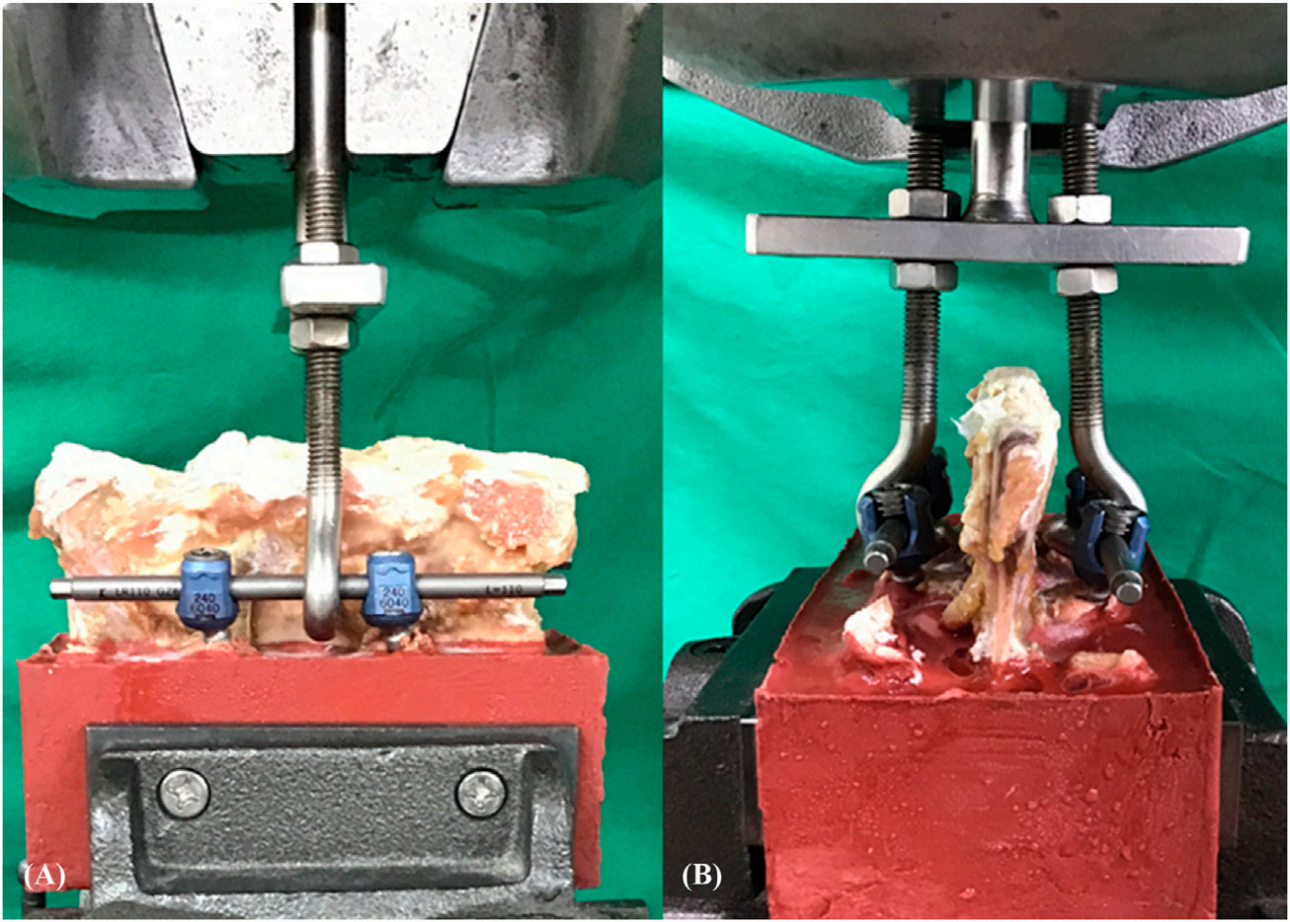
Experimental setup of the screw pullout test of FSU constructs. **(A)** Sagittal view and **(B)** axial view. A custom-made turnbuckle fixture was used to attach the actuator to the rod.

Following the stiffness test, the FSUs were re-embedded into acrylate resin to allow clamping during the construct pullout test (Bionix 810; MTS Systems Corp., MN, United States). A custom-made turnbuckle fixture was used to attach the actuator to the rod (Figure 6). The pedicle screw-rod construct was tested in tension at a rate of 5 mm/min. Load and displacement data were collected at 1.6 Hz. During the pullout test, the relation between the applied force and displacement was simultaneously recorded in 0.1 mm increments until failure. The peak force recorded was defined as the maximal pullout strength for comparison.

### 2.3 Statistical analysis

To evaluate the biomechanical performance of the three trajectory groups in each model, the magnitudes of insertional torque and ultimate pullout force of single vertebrae and stiffness and ultimate pullout force of FSUs were statistically compared. All of the measurements are expressed as the mean ± standard deviation (SD). Statistical software (SPSS for Windows version 12.0, SPSS, Inc., Chicago, IL) was used to analyze the biomechanical performance of all groups in the two models. ANOVA with *post hoc* analyses was performed to evaluate the differences among groups. Differences were considered to be significant at *p* < 0.05.

## 3 Results

### 3.1 Specimen characterization

An appropriate screw trajectory and insertional depth were confirmed using axial and sagittal X-ray imaging prior to biomechanical testing (Figures 2, 3). In the axial view, the pedicle screws in the TT and MT groups were convergently inserted into the vertebral body, whereas in the CBT group, the screw was inserted divergently toward the lateral cortex. In the sagittal view, the screw in the TT group was parallel to the superior endplate; in the MT group, the screw was inserted toward the anterior inferior edge of the vertebral body with proper depth. In the CBT group, the screw was placed 25° caudally toward the superior endplate. No fractures or lateral wall breach were detected in either view.

### 3.2 Biomechanical performance

**FIGURE 7:**
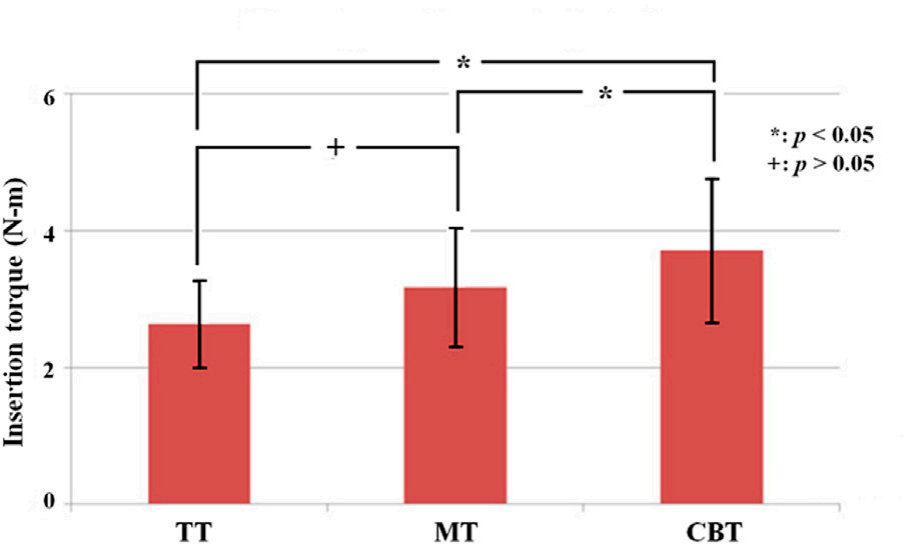
Mean maximal insertion torque of pedicle screws for a single vertebra treated with TT, MT and CBT trajectories. The insertion torque was significantly higher in the CBT group than in the TT and MT groups. Significant difference was not observed between the TT and MT groups.

**FIGURE 8:**
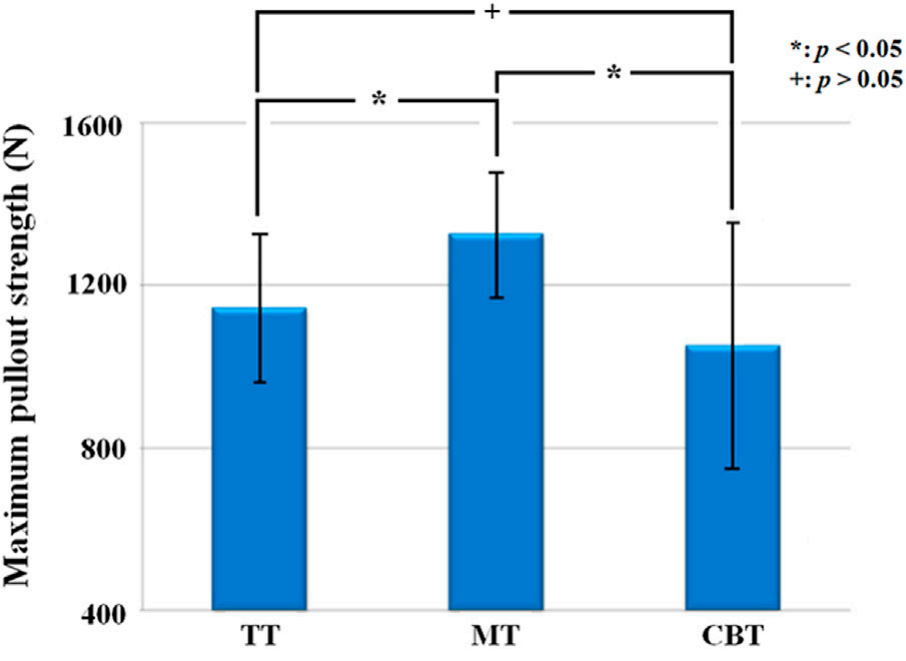
Mean maximum pullout strength of pedicle screws for a single vertebra treated with TT, MT and CBT trajectories. The maximum pullout strength was significantly higher in the MT group than in the TT and CBT groups. Significant difference was not observed between the TT and CBT groups.

In single vertebrae, the insertion torques of the TT, MT and CBT groups were 2.64 ± 0.64, 3.18 ± 0.87, and 3.71 ± 1.05 Nm, respectively (Figure 7). The insertion torque was significantly higher in the CBT group than in the TT and MT groups. There was no significant difference in insertion torque between the TT and MT groups. The maximal pullout strengths of the TT, MT and CBT groups were 1,143.75 ± 181.41, 1,324.69 ± 154.37, and 1,051.61 ± 303.12 N, respectively (Figure 8). The maximal pullout strength was significantly higher in the MT group than in the TT and CBT groups. There was no significant difference in pullout strength between the TT and CBT groups.

**FIGURE 9:**
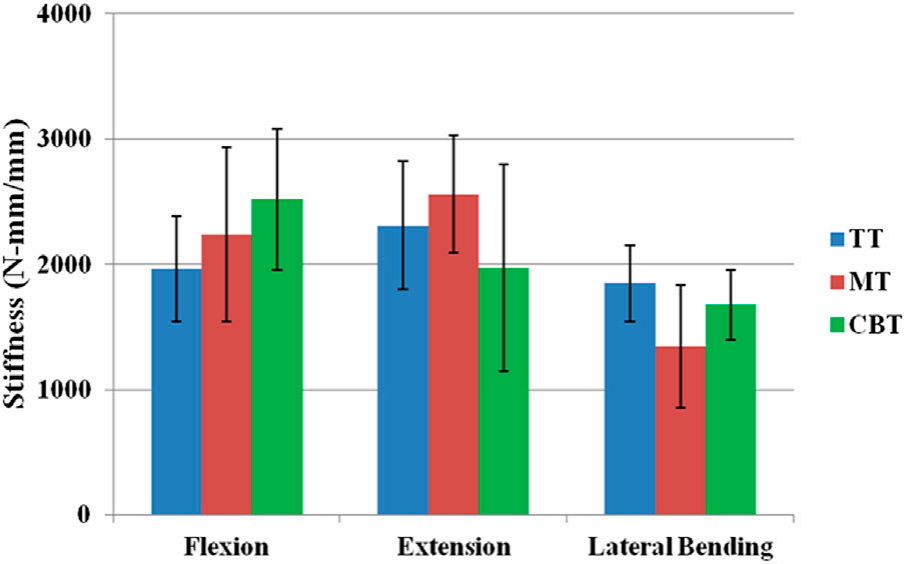
Mean flexion, extension and lateral bending stiffnesses of FSU constructs treated with TT, MT and CBT trajectories. Significant differences were not observed among the groups in the flexion, extension and lateral bending motions (*p* > 0.05).

**FIGURE 10:**
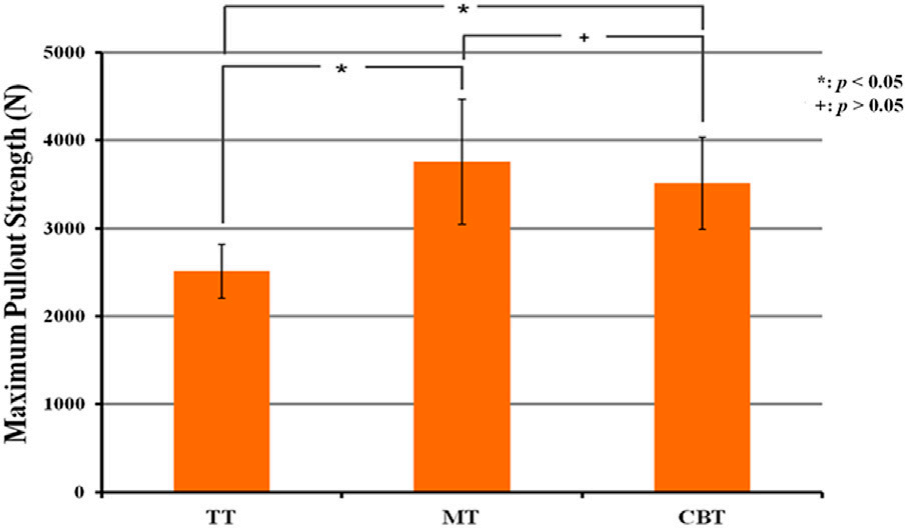
Mean maximum pullout strength of pedicle screws for FSU constructs treated with TT, MT and CBT trajectories. The values in the MT and CBT groups were significantly higher than those in the TT group. There was no significant difference between MT and CBT groups.

In the FSU groups, the stiffnesses of flexion, extension and lateral bending were 1962.9 ± 417.4, 2,307.6 ± 512.1 and 1,847.9 ± 306.9 N-mm/mm in the TT groups, 2,234.7 ± 694.1, 2,558.7 ± 467.6 and 1,344.9 ± 491.9 N-mm/mm in the MT groups and 2,518.4 ± 561.5, 1,968.7 ± 824.5 and 1,675.7 ± 282.1 N-mm/mm in the CBT groups (Figure 9). There was no significant difference in stiffness in the three motions among all groups. The maximal pullout strengths in the FSUs of the TT, MT and CBT groups were 2,511.7 ± 309.2, 3,755.1 ± 711.4, and 3,512.2 ± 519.9 N, respectively (Figure 10). The values in the MT and CBT groups were significantly higher than those in the TT groups. There was no significant difference between the MT and CBT groups.

## 4 Discussion

The aim of this study was to compare the pullout strength and insertion torque of three trajectory methods (TT, MT, CBT) in porcine vertebrae. In single vertebrae, we found the insertion torque was significantly higher in CBT groups than in TT and MT groups (*p* < 0.05). However, the maximal pullout strength was significantly higher in MT groups than in TT and CBT groups (*p* < 0.05). In functional spinal units (FSUs), no significant difference was found in stiffness in the three motions among all groups. The maximal pullout strength in FSUs of MT and CBT groups were significantly higher than the TT groups (*p* < 0.05). Our results demonstrated that either MT or CBT provided better biomechanical performance than TT in single vertebrae or FSUs.

There are three different screw trajectory techniques of the lumbar spine at present. However, there is still no clear consensus regarding the optimal screw trajectory in single vertebrae and functional spine units (Lehman et al., 2003; Phan et al., 2015; Delgado-Fernandez et al., 2017; Hsieh et al., 2021a). Three key factors are responsible for the biomechanical performance of pedicle screw fixation: screw mechanical characteristics, bone quality and implantation techniques (Shen et al., 2019; Matsukawa et al., 2021; Qiu et al., 2022). In our study, bone quality, pilot methods and screw morphometric data, including thread type/shape and outer/inner diameter, were controlled; only the lengths of the three methods were different because of the anatomical characteristics (Lehman et al., 2003; Phan et al., 2015; Delgado-Fernandez et al., 2017; Hsieh et al., 2021a). Newcomb, et al. comparing varying pedicle screw orientations in the axial and sagittal planes on non-idealized finite element vertebrae found that angulations in both the sagittal and axial planes affected stresses on the cortical and cancellous bones and the screw (Newcomb et al., 2017). Pedicle screws placed laterally in the axial plane and superiorly in the sagittal plane reduce the risk of screw loosening and screw breakage. The result implied better biomechanical performance in TT than MT method which is different from our study. Uncontrolled specimens using vertebrae from seven human cadaveric spines and assessment using maximal and cumulative stress on the screw and bone made drastic conclusions. A biomechanical study compared traditional *versus* anatomical trajectory techniques and concluded that the traditional technique results in a 39% increase in maximum insertion torque and a 27% increase in pullout strength compared to the anatomic technique (Lehman et al., 2003). However, cadaveric thoracic vertebrae, various bone density specimens, screws of the same size and uncontrolled anatomical trajectory angles seem clinically impractical. To reveal the true clinical condition without interference by the anterior cortex, the MT tract was longer than the TT tract, so we chose an incremental 5 mm of screw length in the MT groups. The maximal pullout strength was significantly higher in the MT groups than in the TT groups, but there was no significance in insertion torque due to the greater amount of cancellous bone purchased in our study (Hsieh et al., 2020). Varghese, et al. compared the effect of various insertion angles on the pull-out strength in single screw and two screw-rod configurations in normal and osteoporotic polyurethane foam models (Varghese et al., 2017; Varghese et al., 2018a; Varghese et al., 2018b). The pull-out value decreased by 52% from insertion angles of 0°–30° in the single screw configuration. In the two screw-rod configuration, the pull-out strength was maximum for relative lower insertion angles of 10°–15°. The phenomenon was conflict with our study which significantly higher pullout strength was found in higher insertional angle group (MT groups). In our study, for the pullout test using single vertebrae, the screw head was fixed to a custom-made adapter equipped with a universal joint to ensure the long axis of the screw was coaxial with the movement of the testing machine actuator. The experimental setup was different with the above studies (Krishnan et al., 2016; Varghese et al., 2017; Varghese et al., 2018a; Varghese et al., 2018b). In the biomechanical performance of FSUs, motion stiffness of flexion, extension and lateral bending was used instead of direct pullout test from construct rod. Studies retrospectively analyzed the anatomical location of acute thoracolumbar vertebral fractures and found that the fracture rate of the superior endplate is much higher than that of the inferior endplate (Che-Nordin et al., 2018; Wang et al., 2020). Our biomechanical results suggested that MT screws are not only superior in pullout strength but also potentially decrease future upper instrumented fractures.

CBT involves a medial-to-lateral and a caudo-cephalad direction from the pars interarticularis with the objective of maximizing thread contact with the higher-density cortex. The fixation mechanism was totally different with the TT or MT group, so we chose a shorter-sized and fine threads screws as commonly used screws in the clinic. The insertion torque was significantly higher in the CBT groups than in the TT and MT groups, which may be due to the fine threads cortex fixation (Matsukawa et al., 2013; Oshino et al., 2015); however, there was no significant difference in maximal pullout strength, this may attributed to the shorter fixation length of screws in CBT. In a 124-patients prospective randomized controlled trial comparing CBT and TT fixation method for osteoporotic single-level lumbar fusion showed significantly better post-operative lumbar stability in the CBT group (Ding et al., 2022). Both the pullout strength and insertion torque showed higher values in the CBT group than in the TT group when using a diseased osteoporotic spine to highlight the fine threads effect in the CBT groups (Santoni et al., 2009; Baluch et al., 2014; Matsukawa et al., 2014; Matsukawa et al., 2015a; Matsukawa et al., 2015b).

In our stiffness test of FSUs, the horizontal pin and vertical motion path of the specimen resulted in a *3-D* configuration that ensured that the specimen moved vertically as the spinal construct was flexed, extended or laterally bent, which excluded other biomechanical interfering factors during setting up. To the best of our knowledge, no study has evaluated FSUs and compared intervertebral stability among the three trajectory methods. Our biomechanical comparison between TT and CBT corresponded to other studies (Perez-Orribo et al., 2013; Oshino et al., 2015). Perez-Orribo et al. (Perez-Orribo et al., 2013) found that CBT demonstrated equivalent stiffness to TT in bending tests of human cadaveric lumbar FSUs with presence of interbody fusion. Oshino et al. (Oshino et al., 2015) also mentioned the same phenomenon in deer lumbar FSUs.

In our present study, a higher pullout strength of FSUs was exhibited in the MT and CBT groups than in the TT group without significant differences in stiffness, indicating that the longer screws in the MT group and larger cortical contact in the CBT group increased the efficacy of the screw. However, the lack of significant differences in stiffness among the three groups may be due to different spinal mobility between quadrupeds and humans (Hart et al., 2006; Cornaz et al., 2021) and multiple anatomical confounding factors, including facet joints, disco-vertebral joints and interspinous ligaments (Putzer et al., 2016; Bashkuev et al., 2018).

The limitations of this study include the following: first, porcine spines were used as specimens, and different geometries or density distributions of vertebrae might affect the generalizability of our results to human patients (Schomberg et al., 2016; Miranpuri et al., 2018). CT scans found that similar vertebral body height, shape of the end-plates, shape of the spinal canal, and pedicle size between human and porcine spine (Busscher et al., 2010). The size of both superior and inferior endplates increased more caudally in the human spine but less increased in porcine spine, which indicate the vertebral width in human was larger than porcine. More purchase of CBT screws would be expected in human spine which implied the significantly higher value of insertion torque would be achieved in the CBT group than in the TT and MT groups in single human vertebrae; and the maximal pullout strength could be corresponding to the insertional torque. Second, screws of more sizes and different geometries including cannulated, conical, or dual threaded pedicle screws should be performed to obtain a more conclusive result. Third, the limited number of specimens might increase the variability of the data and reduce the statistical reliability. Finally, the lack of significance in motion among the three methods implies that intervertebral stiffness could be affected not only by pullout strength but also by more functional spinal units, which should be performed for further clinical application.

## 5 Conclusion

The *in vitro* biomechanical study provides insight into the fact that MT can provide a higher pullout strength and CBT can provide a higher insertion torque in single vertebrae. In FSUs, the higher pullout strength found in the MT and CBT groups corresponded to the single vertebrae. The lack of significance of stiffness in one-level FSUs among the three methods suggested that MT and CBT could both be reasonable alternatives to TT if the traditional tract was not feasible.

## Data Availability

The original contributions presented in the study are included in the article/Supplementary Material, further inquiries can be directed to the corresponding author.
